# Why does the immune system of Atlantic cod lack MHC II?

**DOI:** 10.1002/bies.201200005

**Published:** 2012-04-13

**Authors:** Bastiaan Star, Sissel Jentoft

**Affiliations:** Centre for Ecological and Evolutionary Synthesis (CEES), Department of Biology, University of OsloOslo, Norway

**Keywords:** adaptive immunity, Atlantic cod, genomics, innate immunity, MHC

Basic features of the vertebrate adaptive immune system have been conserved since their emergence in the ancestral lineage that lead to all jawed vertebrates 1, 2. Atlantic cod (*Gadus morhua*) is the first vertebrate reported to have lost one of these features, the antigen presenting major histocompatibility complex (MHC) II system 3. This system is responsible for the classical adaptive immune response against bacterial and parasitic infections through the activation of CD4^+^ T cells in other vertebrates, and defects in MHC II functionality are in general considered to lead to severe immunodeficiency and death. Therefore, a functional and evolutionary understanding of this phenomenon could uncover novel immunological functionality and elucidate basic selective mechanisms affecting vertebrate immune systems.

## Genetic drift or directional selection?

The evolutionary context in which the unusual immune system of Atlantic cod evolved is unclear, though two different scenarios may explain its existence. The first scenario hypothesizes that functionality of major histocompatibility complex (MHC) II system is not crucial for the immune defense in teleosts, and that the loss in Atlantic cod has been predominantly driven by genetic drift, rather than by specific biological or environmental factors. The second scenario hypothesizes that somewhere in the evolutionary past the function of the MHC II has been lost through directional selection. An important assumption of such a directional selection scenario is that some biological or environmental factors have driven this process, in contrast to the first scenario whereby no such association is expected. Within the directional selection scenario, we here consider two alternative selection hypotheses that separately or in combination could have lead to the loss of MHC II. First, *the metabolic cost hypothesis* suggests that despite being of some adaptive value, the benefit of MHC II functionality was in some environments not sufficient to compensate for the metabolic costs of expressing this system. Thus, directional selection would favor mutations that disrupt the expression of this system, resulting in the eventual loss of genes underlying its function. Second, *the functional shift hypothesis* suggests that in some environments functionality of other immune system components than MHC II, either belonging to the innate or adaptive immune system, provided a more flexible alternative evolutionary immunological strategy compared to the functionality provided by the MHC II system. This strategy may then have given better adaptation to certain environmental challenges than MHC II, making the system obsolete and exposed to genetic drift. It may also be that further evolution of such alternative strategy required dismantling of the MHC II system for mechanistic reasons and thus caused a selection pressure against its presence. Below we assess the likelihood of each of these scenarios and selection hypotheses using available empirical data and elaborate on investigations aimed to separate them.

The genetic drift scenario appears implausible from a mammalian perspective. However, fundamental differences in genomic organization and functionality of the immune system have been found in teleost lineages. For example, in contrast to mammals, no conserved linkage exists in teleosts between genes for MHC I and II 4, and this absence could facilitate independent evolution of these systems. Moreover, components of the innate immune system show greater structural and functional diversity in teleosts while adaptive components are restricted, for example through a more limited antibody repertoire 5. Because their adaptive immune system matures relatively late in ontogeny, teleosts also initially rely on innate responses during their extrauterine development 6. MHC II functionality might therefore not be critical in teleosts and relatively easily lost through genetic drift. If MHC II function is not critical, its loss is expected in a wide variety of teleosts with no ecological or evolutionary relationships with Atlantic cod. Despite reports of unusual immune systems in sea horse and pipefish, widespread loss of MHC II however, does not appear to be prevalent among teleosts 7, 8. Thus, a pure genetic drift scenario appears insufficient to explain the loss of the MHC II system.

## Lack of MHC II coincides with a distinct immune gene repertoire

**Figure 1:**
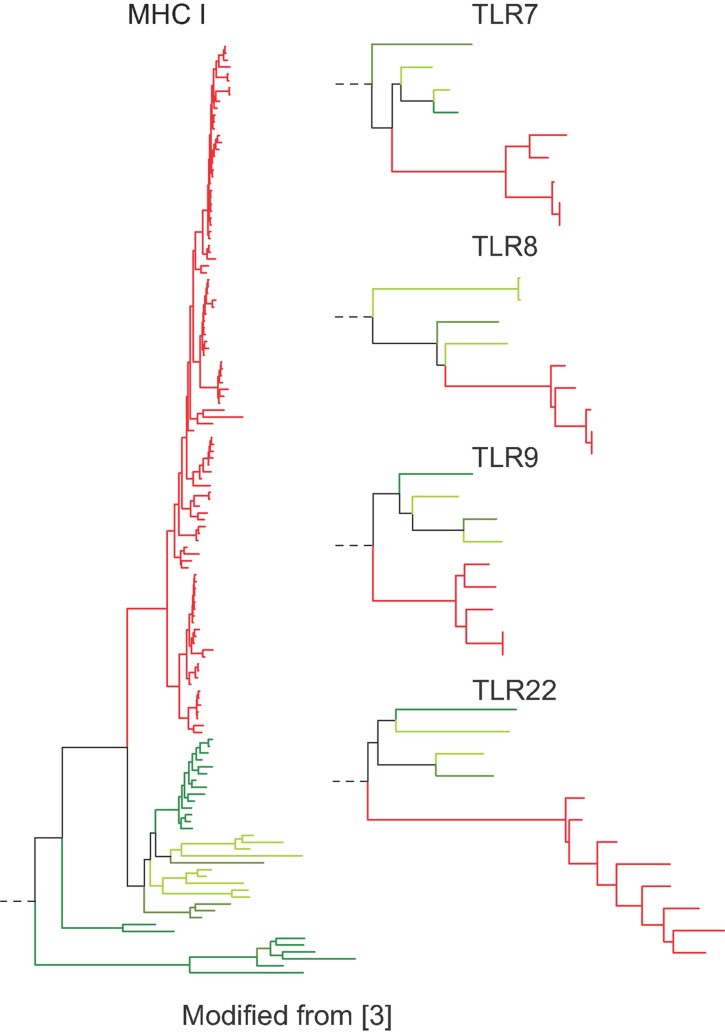
The lack of MHC II in Atlantic cod coincides with evidence of positive selection through the expansion of genes for MHC I and several Toll-like receptor families. These expansions are symbolized by the extensive tree topology of Atlantic cod (red) compared to that of other teleosts (shades of green).

The metabolic cost and functional shift hypotheses regard the architecture underlying immune functionality fundamentally different. The metabolic cost hypothesis proposes that MHC II functionality can be lost independently from other changes in the immune system, whereas the functional shift hypothesis proposes that functionality of other immune components has to expand through directional selection as a prerequisite for this loss. Can we find evidence in the gene repertoire of Atlantic cod for either hypothesis? Interestingly, evidence for directional selection is found in several gene families underlying both adaptive and innate immune systems in Atlantic cod through expansions of genes for MHC I and Toll-like receptors (TLRs) 3, indicative of enhanced functionality for these systems (Fig. 1). The expansion of the TLRs is particularly intriguing. High copy numbers of TLRs have also been reported in evolutionary distant lineages to Atlantic cod, such as invertebrates (sea urchin, 222 TLRs) and chordates (amphioxus, 77 TLRs), and these numbers have been discussed in relation to the absence of a specialized adaptive immune system in these species 9–11.

While the function of these TLRs is still unknown, the increased number of TLRs in Atlantic cod highlights the possible existence of a fundamental trade-off between adaptive and innate capabilities whereby a stronger innate system may, to some extent, lead to reduced dependence on the adaptive system 12. Functional studies on the specificity of the Atlantic cod immune response appear to support such a view. Initially, specific antibody responses were considered poor in Atlantic cod 13 and this poor immune response was subject of debate 14. While specific antibody production has been detected in more recent experiments 15, vaccination also proved effective in absence of a measurable specific immune response 16, suggesting that mechanisms other than the classical adaptive immune response provide protection.

The indication that particular components of the Atlantic cod have experienced positive selection appears to support the functional shift hypothesis. Nevertheless, these changes may have evolved after MHC II was lost, and merely incidentally coincide with this phenomenon. They are therefore not direct evidence that rejects the metabolic cost hypothesis. Moreover, the metabolic cost and functional shift hypothesis can be difficult to distinguish retrospectively if they have affected the immune system simultaneously. If metabolic costs have been the predominant evolutionary force affecting the loss of MHC II however, then teleost lineages may exist in which MHC II functionality is absent, while no other functional changes have coincided with this loss. Following similar reasoning, if the emergence of alternate immune functionality occurred before the loss, lineages may exist that have such functionality and in addition have an MHC II system in different stages of disarray. In fact, the functional hypothesis proposes that the phenomena observed in the Atlantic cod genome are tightly coupled. The different assumptions underlying the metabolic cost and functional shift hypotheses therefore predict distinct gene repertoires underlying the architecture of immune systems among the teleost lineages.

## Historical ecological settings

Each of the directional selection scenarios assumes that definitive factors, whether biological or environmental, can be associated with this distribution. Interestingly, in the current ecological setting, no biological or environmental factors specific to the habitats of Atlantic cod are easily identifiable. Indeed, this species lives among teleosts in which genes for MHC II have been identified and which presumably rely its functionality. Moreover, the characteristics of the Atlantic cod immune system appear shared within the gadoid lineages. For example, burbot (*Lota lota*) is a gadoid that lives in fresh water and lacks the same set of genes involved in the MHC II system in expressed transcripts 3. These findings indicate that its peculiar immune system evolved far back in the evolutionary past and that the current ecological setting of Atlantic cod may not reflect the one in which this system originally evolved.

Atlantic cod belongs to the family of Gadidae, within the order Gadiformes for which fossil evidence exists as early as 65 million years BP 17. Interestingly, several families (i.e. Macrouridae, Bathygadidae, Muraenolepdidae) inside the order Gadiformes contain deep-sea species that populate the bathypelagic or bathybentic regions of the ocean 18. Thus, the gadoids may have evolved from a teleost lineage with a deep-sea habitat, which is an environment that is relatively cold, and has a different microbial community compared to that of mesopelagic or epipelagic environments. This community is characterized by a larger proportion of archaea and a lower microbial abundance with increasing depth 19. Both temperature and composition of the microbial community are considered important drivers in the evolution of the vertebrate immune system 5, 20–22. Temperature strongly influences metabolic rates in poikilothermic organisms and low temperatures modulate the immune system so that adaptive parameters become weaker and innate parameters become stronger 5, 23. Thus, the combination of low temperature and a characteristic microbial community may have promoted an immune system with a relative strong innate component and a weaker adaptive component, resulting in the loss of the MHC II system.

## Comparative genomics

How can we investigate the validity of the metabolic cost and functional shift hypotheses and associate specific biological and environmental factors with the directional selection scenario? Of course, it would be optimal to obtain a thorough functional understanding of the adaptive and innate immune systems in divergent teleost lineages. Such understanding is not achievable in the near future, in particular for those lineages without commercial value. Nevertheless, the selection scenario predicts particular distributions of immune architecture among divergent teleosts. For example, if low temperatures have been a major factor in the loss of MHC II and the coinciding functional changes for MHC I and the TLRs, we can expect analogous immune systems in lineages that have adapted independently to a cold environment, such as the Antarctic ice fishes (Notothenioidei). The ice fishes may provide a particularly interesting comparison: A relatively high parasitic load has been reported in these species and Atlantic cod 24, 25. Moreover, sequence analysis of ice fish immunoglobulins has shown some unexpected features such as a relatively long hinge peptide 25. Nevertheless, a complete genome has not yet been obtained for these species.

By characterizing the complete immune gene repertoire in a wide range of teleosts from specific environments, we can infer which of the two alternative hypotheses is more likely. These investigations will then also illuminate the strength of a potential trade-off between adaptive components like MHC II and putative functional changes in immune architecture, which may be a fundamental characteristic of vertebrate immune systems. A full comparative analysis of the immune gene repertoire of multiple species is rapidly becoming economical, considering the ongoing reduction in sequencing costs.

## Host-microbial interactions

A central question within comparative immunology remains whether environmental factors like temperature or biological factors like host-microbial co-evolution are the most important drivers in vertebrate immune evolution. Host-commensal bacterial co-evolution recently received substantial attention, e.g. reduced commensal loads in the intestines of invertebrates could be associated with an absent adaptive immune system 26. Interestingly, host-commensal associations in mammalian intestines may be extensively regulated by the CD4^+^ T cells 27 that are absent in Atlantic cod. It is therefore relevant to investigate whether the interactions between Atlantic cod and its microbial community are different from those in ecologically comparable teleosts with a functional MHC II system. So far, the intestinal community of Atlantic cod appears comparable to that of other teleosts 28, 29, but a full metagenomic analysis of its microbial community in natural conditions remains to be done. Interestingly, a specific core microbial gut community has been observed in zebrafish, despite substantial differences in rearing conditions 30. An observation of a unique core microbial community that may be composed of specific bacteria or employ particular biochemical signaling cascades in teleosts without MHC II functionality would support the idea of an important role for host-microbial interactions for the evolution of vertebrate immunity.

## Conclusions

Here, we have discussed several scenarios and selection hypotheses for the loss of functionality of MHC II and the emergence of the unusual immune system of Atlantic cod. It remains to be determined whether a metabolic cost or functional shift hypothesis has been predominantly responsible, and which specific biological and environmental factors have driven these selection scenarios. Nevertheless, without doubt, the existence of this peculiar immune system provides a window of opportunity for studying fundamental evolutionary questions central to our understanding of vertebrate immunity.

## Abbreviations

MHC: major histocompatibility complex
TLR: Toll-like receptor
